# Facile fabrication of self-assembled nanostructures of vertically aligned gold nanorods by using inkjet printing†

**DOI:** 10.1039/d1ra03900h

**Published:** 2021-06-24

**Authors:** Koichiro Saito, Keegan McGehee, Kengo Manabe, Yasuo Norikane

**Affiliations:** Research Institute for Advanced Electronics and Photonics, National Institute of Advanced Industrial Science and Technology (AIST) Higashi 1-1-1 Tsukuba Ibaraki 305-8565 Japan koichiro.saito@aist.go.jp; Department of Chemistry, Faculty of Pure and Applied Sciences, University of Tsukuba Ibaraki 305-8571 Japan

## Abstract

We demonstrated that the vertically aligned gold nanorods (AuNRs) were quickly and easily formed by using inkjet printing when aqueous dispersion of AuNRs containing a small amount of ethylene glycol (EG) was employed as an ink. It was observed that the content of EG in water suppressed rapid drying and convection in the droplets, which is favorable for the formation of the nanostructures.

## Abstract 2

Slow evaporation of a droplet of water/ethylene glycol (EG) mixture allows the fabrication of vertically aligned gold nanorods using inkjet printing.

## Graphical abstract

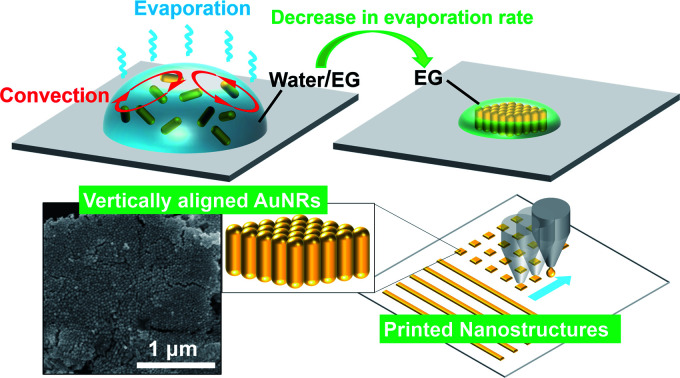

Self-assembly is a useful technique for improving the functionality of colloidal nanomaterials.^1–3^ For example, when nanoparticles such as gold and silver, which exhibit localized surface plasmon resonance, form self-assembled nanostructures, the plasmon resonance will be further enhanced.^4–7^ In recent years, nanostructures in which gold nanorods (AuNRs) are vertically aligned on a substrate have attracted particular attention.^8–11^ In such nanostructures, strong plasmon resonance appears uniformly in the gaps between the particles. Therefore, they can be applied to photoluminescence enhancement^12–14^ and reproducible surface-enhanced Raman scattering (SERS) sensing.^15–18^

However, strict fabrication conditions make it difficult to put the vertically aligned AuNRs into practical use. For preparation of vertically aligned AuNRs on a substrate, it is basically necessary to slowly evaporate a droplet of the dispersion of colloidal AuNRs for about a day while keeping the droplet under high humidity conditions.^8^ While effective, the fabrication process is very time-consuming. Although rapid fabrication is possible by using AuNRs dispersed in an organic solvent, a complicated process of ligand exchange is required.^19^ In addition, when drying a large-sized droplet on a substrate, it is difficult to precisely control the position where the structure will be formed. Thus, for practical use, a technique that enables rapid fabrication of vertically aligned AuNRs in any pattern at any positions on a substrate is absolutely necessary. One promising method is using inkjet printing technology (Fig. 1(a)), which has been used to make self-assemblies of nanomaterials such as polystyrene nanoparticles.^20^

**Fig. 1:**
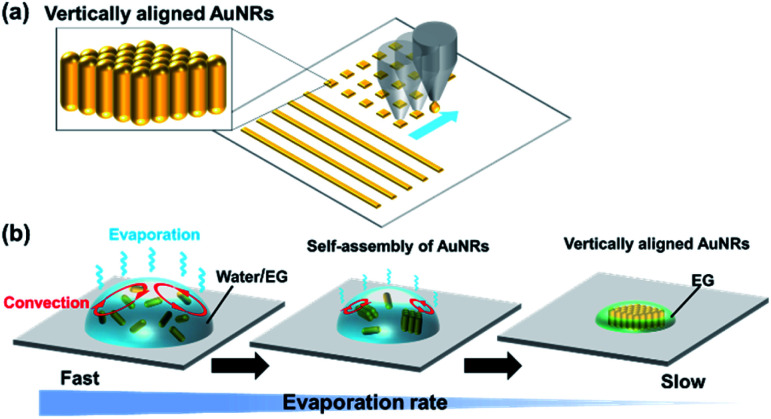
(a) Schematic illustrations of the vertically aligned AuNRs fabricated by inkjet printing. (b) The formation of the vertically aligned AuNRs in the droplet of the water/ethylene glycol (EG) mixture during evaporation.

Inkjet printing has received a great deal of attention in recent years because it can accurately deposit materials at any location. It allows the printing pattern to be changed without the use of printing plates or photomasks, which leads to lower costs and lower material consumption. Therefore, it is widely used in the manufacture of electronic circuits,^21^ sensors,^22^ thin film transistors,^23^ solar cells^24^ and light emitting diodes.^25^ If vertically aligned AuNRs can be rapidly prepared by using inkjet printing, a facile construction of chip-based platforms for biosensing and SERS sensing will be enabled. However, in the inkjet printing process, the solvent of the ink dries at a high speed, which is the opposite of the conditions for the formation of the vertically aligned AuNRs. This may be the reason why the inkjet printing has not been employed previously. Here, we attempted to fabricate vertically aligned AuNRs by inkjet printing using an aqueous dispersion of AuNRs mixed with a low-volatility solvent as an ink.

Much research is still being done to elucidate the formation mechanism of vertically aligned AuNRs. It is known that van der Waals attraction, electrostatic repulsion, and depletion forces between the AuNRs affect the formation of the structure. It has also been confirmed that slow evaporation of the droplet is a particularly important factor.^18,26^ Recently, Jung *et al.* have reported favorable conditions for the formation of vertically aligned nanorods by numerically solving the Smoluchowski coagulation equation under moving boundary conditions.^27^ According to the report, the condition where the evaporation rate is slow at a sufficiently high concentration of nanorods in the droplet promotes the formation of the nanostructure. This finding suggests that the droplets do not need to evaporate slowly during the entire drying process. Since the concentration of nanorods increases as the droplets evaporate, the evaporation rate needs to be slow just before the droplets are completely evaporated. If this condition is satisfied, the formation of vertically aligned AuNRs can be expected even by using inkjet printing, in which the ejected fine droplets are quickly dried. Therefore, we proposed to use an aqueous dispersion of AuNRs containing a small amount of a low-volatility solvent. It was expected that the formation of the vertically aligned AuNRs would be assisted by the slow evaporation of the low-volatile solvent after the rapid evaporation of water (Fig. 1(b)).

We used ethylene glycol (EG) as the low-volatility solvent. First, we observed the drying behavior of droplets of the mixed solution with an optical microscope. 0.4 M EG aq. was prepared and 0.5 μL of the solution was dropped onto a cleaned silicon substrate ((100) surface). The surface of the silicon substrate was cleaned by a UV ozone cleaner. The temperature was around 25 °C and the relative humidity was around 30%. Fig. 2(a) shows how the size of the droplet decreases due to the evaporation. The diameter of the droplets decreases rapidly in the first 6 minutes. Then, the decrease rate clearly slowed down. The drying behaviors of the EG/water mixture, pure water and pure EG droplets are shown in Movies S1, S2 and S3 (see ESI†), respectively. Fig. 2(d) shows the change in the projected area of each droplet. The droplets of the EG/water mixture evaporated in the same way as pure water for the first 6 minutes (Fig. 2(b)). After that, the evaporation became very slow, similar to the evaporation behavior of pure EG (Fig. 2(c)). This suggests that in the evaporation of the EG/water mixture, the remaining EG slowly evaporates after the rapid evaporation of water. This behavior is expected to be a favorable condition for the formation of vertically aligned AuNRs.

**Fig. 2:**
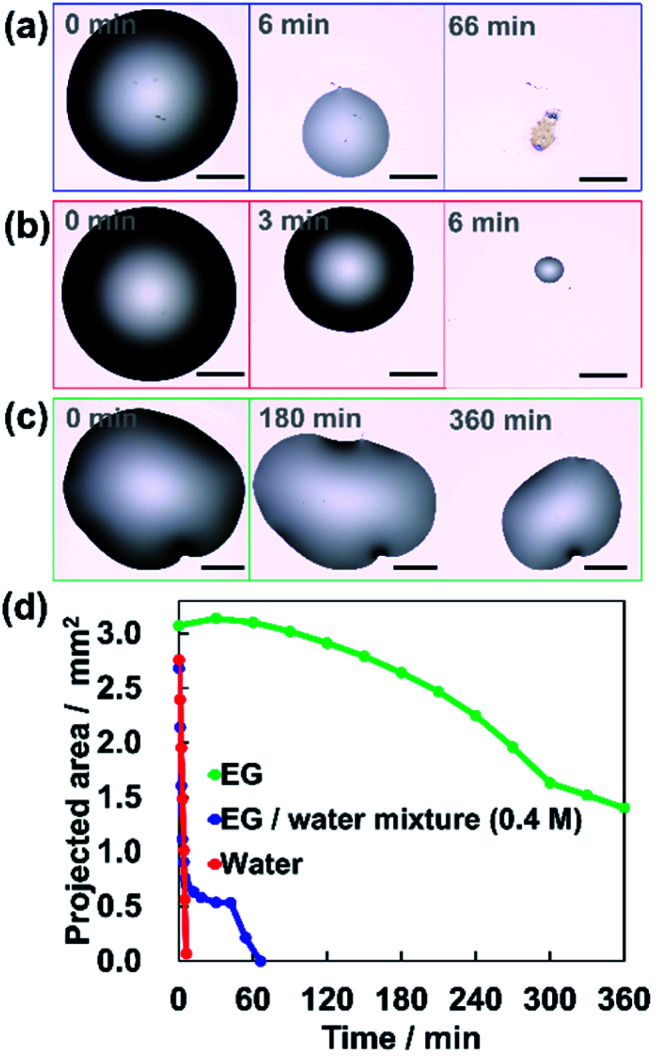
Microscopic images of the drying behavior of (a) EG/water mixture, (b) water, and (c) EG. The scale bars are 500 μm. (d) The changes in the projected area during the evaporation for each droplet of EG/water mixture, water, and EG.

In the process of evaporation, convection is driven in the droplet.^28^ It has been reported that suppression of this convection facilitates the formation of vertically aligned AuNRs.^18^ Thus, we performed particle image velocimetry (PIV) analysis to observe the flow driven in the droplets on the substrate. Fig. 3(a) shows the vector field of the average flow velocity of tracer particles (fluorescent pigment, particle size 3–5 μm, JUJO Chemical Co., Ltd.) in a droplet of pure water. The frame rate was 50 fps and the measurement time was 110 seconds. The convection due to evaporation was driven from the edge to the center of the droplet. The motions of the tracer particles can also be observed in Movie S4.†

**Fig. 3:**
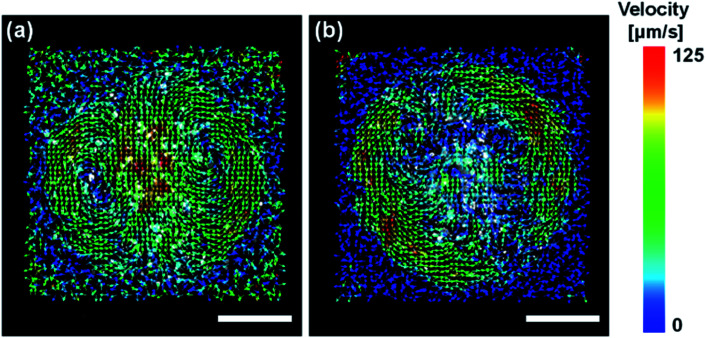
The convections driven in the droplets for (a) water and (b) EG/water mixture during evaporation. The arrows show the average velocity of flow at each point analyzed by particle image velocimetry (PIV). The color bar indicates the magnitude of the velocity. The scale bars are 100 μm.

Fig. 3(b) shows the vector field of the average flow in the droplet of 0.4 M EG aqueous solution. Although a flow appeared at the edge of the droplet, there was no clear flow near the center. As one can observe in Movie S5,† convection initially carries particles throughout the droplet. However, once the contact line begins to retract due to evaporation the convection velocities approach zero. The particles showed chaotic movement around the center, which was not recognized as a flow in a specific direction detected by PIV. Faster evaporation of water at the air-liquid interface would be expected to cause local increase in the EG concentration, resulting in the suppression of the motion of the particles from the edge to the center. From these results, it was found that the EG in the droplet suppressed the convection, which is a favorable condition for the formation of vertically aligned AuNRs.

With evidence to suggest its potential, we attempted to fabricate vertically aligned AuNRs by using inkjet printing. The AuNRs were synthesized and concentrated to about 3 nM by centrifugation.^29,30^ The average length and width of AuNRs were about 76 ± 9.2 nm and 29 ± 4.1 nm, respectively. The average aspect ratio was about 2.6. The detailed synthetic procedure is described in ESI.† The AuNRs were protected by cetyltrimethylammonium bromide (CTAB) and dispersed in water. The CTAB concentration was fixed at 3 mM for all AuNR dispersions used in this experiment. The EG concentration of each dispersion was adjusted to 0 M, 0.1 M, 0.4 M, and 1.0 M. We used electrostatic inkjet printing (Microjet FemtoJet-2000HB, MICROJET Corp., Fig. S1, see ESI†), which is suitable for the fabrication of micropatterns. Line patterns (∼100 μm width, ∼200 μm interval) as shown in Fig. 4(a) were successfully prepared.

**Fig. 4:**
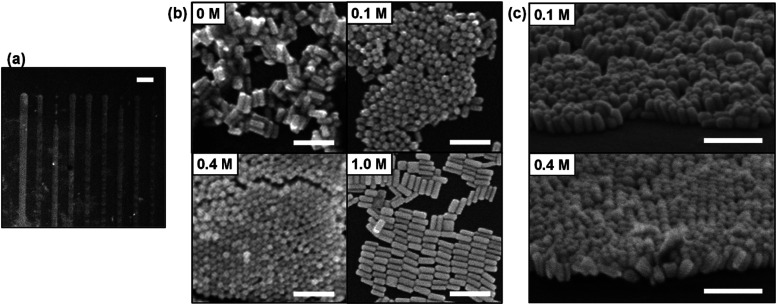
(a) An SEM image of line patterns of AuNRs assembly fabricated by inkjet printing on a silicon substrate. The scale bar is 200 μm. (b) The nanostructures of AuNRs resulting from varied EG concentration in the printing inks, 0 M, 0.1 M, 0.4 M, and 1.0 M. The scale bars are 200 nm. (c) Vertically aligned AuNRs fabricated by using 0.1 M EG and 0.4 M EG inks observed at an angle of 65°. The scale bars are 200 nm.

Fig. 4(b) shows the nanostructures fabricated by inkjet printing of AuNR dispersions at each EG concentration observed by using scanning electron microscope (SEM). The zoomed-out images are shown in Fig. S2(a)–(d).† For 0 M EG dispersion, random aggregation was formed. Vertically aligned AuNRs were observed for 0.1 M and 0.4 M EG dispersion. As shown in Fig. 4(c), when the sample is tilted, AuNRs standing perpendicular to the substrate are observed. This revealed that the addition of low concentration of EG can dramatically facilitate the formation of vertically aligned AuNRs. These results support our hypothesis based on the previous reports.^27^ Furthermore, it was also confirmed that the vertically aligned AuNRs were formed when 10 μL droplets (0.1 M EG or 0.4 M EG) were cast and dried on a silicon substrate under atmospheric conditions (Fig. S3(a) and (b)†). We have achieved the fabrication of vertically aligned AuNRs in a very simple method. In the case of 1.0 M EG dispersion, it should be noted that AuNRs were assembled parallel to the substrate. Similarly, vertically aligned AuNRs could not be observed in the case of 1.78 M EG dispersion (10 vol%) (Fig. S4†). Further showing that when the EG concentration is too high, it becomes difficult to form vertically aligned structures.

We investigated surface-enhanced Raman scattering (SERS) for vertically aligned AuNRs fabricated by using 0.4 M EG dispersion. The substrate was immersed in 1 nM benzyl butyl phthalate (BBP) ethanol solution for several seconds, then allowing it to dry completely in an ambient condition. BBP is a type of environmental pollutant. As shown in Fig. S5,† a peak appeared at 1005 cm^−1^, which is attributed to a β (CC) loop in-plane deformation mode.^31^ Although we have succeeded in detecting the trace amount of BBP, the sensitivity is lower than the previously reported BBP detection using vertically aligned AuNRs.^16^ It is considered that this is because the number of the domains where the AuNRs are vertically aligned is not enough for the printed area. It is expected that the sensitivity will be improved by drawing the pattern multiple times using inkjet printing.

EG is known to have the effect of increasing the critical micelle concentration (CMC) of CTAB.^32^ Therefore, one would expect the addition of EG to the dispersion to destabilize the CTAB bilayers surrounding AuNRs, resulting in random aggregation. The peak wavelength of the absorbance of the dispersion changes significantly according to the aggregation of AuNRs.^33,34^ Thus, the degree of aggregation can be estimated from the spectral shape. For AuNRs dispersions with different EG concentrations, absorbance spectra were measured by using a UV-Vis spectrometer (V-670, JASCO Corp.). Fig. 5(a) shows the absorbance spectra of the AuNRs dispersions with EG concentration of 0 to 1.0 M. There was almost no change in the spectral shape, suggesting that the AuNRs in the dispersions were stable.

**Fig. 5:**
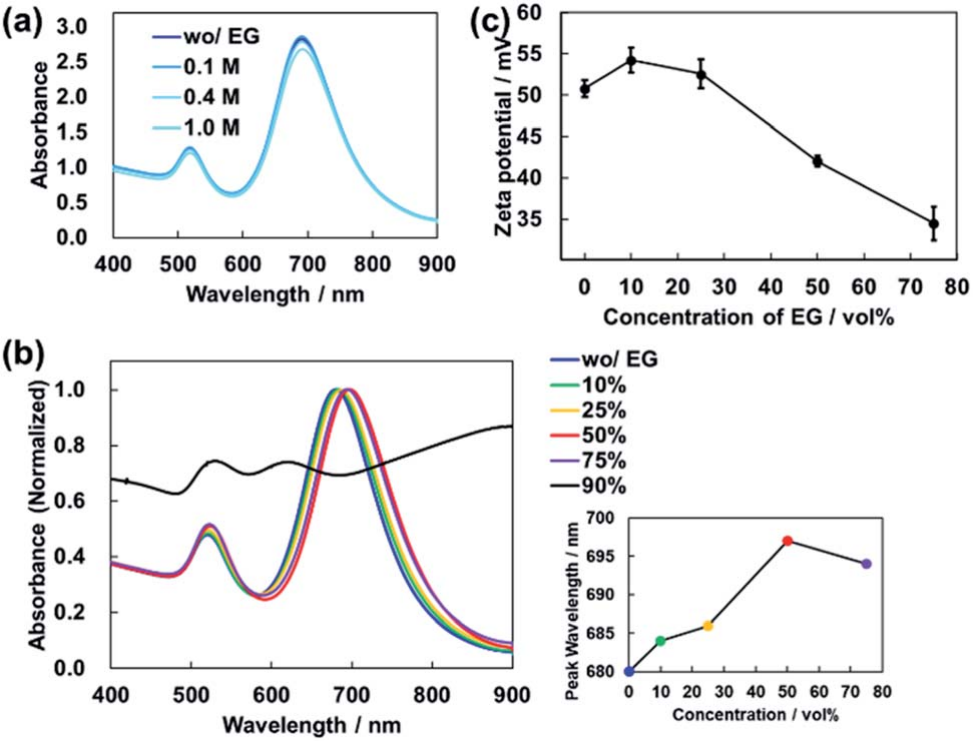
(a) Absorbance spectrum of AuNRs dispersion for each EG concentration, 0, 0.1, 0.4, and 1.0 M. (b) Spectral changes in absorbance with increasing EG concentration, 0, 10, 25, 50,75, 90 vol%. The inset shows the peak wavelength corresponding to each EG concentration. (c) Decrease in the zeta potential value caused by increased EG concentration.

However, in this experiment, the concentration of EG gradually increases as the water in the droplet evaporates at high speed. We also measured the absorbance of the AuNRs dispersions with high EG concentrations. As shown in Fig. 5(b), vol%. The peak wavelength was slightly redshifted (Fig. 5(b) inset). This is because the addition of EG increased the refractive index of the mixture solution. In principle, the peak wavelength of absorbance of AuNRs is redshifted almost linearly according to the refractive index of the dispersion.^29^ For the dispersion used in this experiment, the peak wavelength of absorbance and the refractive index have an almost linear relationship in the range of EG concentration from 0 to 50 vol% (Fig. S6†).

On the other hand, for 75 vol% EG dispersion, the peak was blue-shifted, suggesting that AuNRs were slightly aggregated (Fig. 5(b) inset). As shown in Fig. 5(b), for 90 vol% EG dispersion, the spectral shape changed significantly, resulting in the loss of a clear peak. This indicates that the increase in EG concentration caused aggregation of AuNRs.

Next, we investigated the changes in the zeta potentials of AuNRs due to the addition of EG to the dispersions by using electrophoretic light scattering measurement (Zetasizer ZS, Malvern Panalytical Co., Ltd.). As shown in Fig. 5(c), the zeta potential of AuNRs decreased as the EG concentration increased, which indicates that the AuNR dispersion became unstable. This result is consistent with the result of the absorbance measurement. Note that for 90 vol% EG aqueous mixture, it was impossible to measure the zeta potential because the dispersion did not contain enough particles due to the aggregation.

From these results, it was found that the addition of a small amount of EG did not affect the stability of AuNRs in the dispersion, but aggregation occurred at a high concentration of EG. Sufficiently high concentration of AuNRs in the droplet is required for the formation of vertical structures,^16,18^ which means that the AuNRs concentration relative to the amount of EG is important.

When the concentration of AuNRs in the droplet is high, AuNRs are close to each other, while being electrostatically repelled by CTAB. This reduces orientational degrees of freedom of the AuNRs and their alignment is facilitated.^8,35^ As the volume of the droplet decreases due to evaporation, the aggregation will be caused by the further increase in AuNRs concentration, resulting in the formation of vertically aligned AuNRs. At this time, the slow evaporation rate of the droplets allows the AuNRs to assemble slowly. The slow evaporation rate of EG further facilitates the formation of vertically aligned AuNRs.

On the other hand, as shown in Fig. 5(b) and (c), when the concentration of EG is too high, the aggregation of AuNRs occur due to the decrease in electrostatic repulsion. In a droplet of a mixture of EG and water, the concentration of EG will gradually increase because the water evaporates more quickly. Therefore, when the EG concentration increases to around 90 vol%, the aggregation of AuNRs occurs regardless of the AuNRs concentration. In the droplet with low concentration of AuNRs at this point, the AuNRs are far apart from each other. Thus, AuNRs can be oriented freely, resulting in randomly oriented aggregation.

In the process of droplet evaporation, both EG concentration and AuNRs concentration increase. Since the initial concentration of AuNRs is fixed in this experiment, the AuNRs concentration relative to the amount of EG becomes low when the initial concentration of EG is high. Therefore, it is probable that for EG concentrations of 1.0 M and 1.78 M, the AuNRs concentrations at the start of the aggregation were not high enough for the formation of vertically aligned AuNRs. For EG concentrations of 0.1 M and 0.4 M, the AuNRs concentrations were sufficiently high relative to the amount of EG. In addition, the EG causes slow evaporation when AuNRs start to aggregate. These two conditions, high AuNRs concentration and slow evaporation rate, are critical factors that facilitate the formation of vertically aligned AuNRs. In other words, there should be an optimum value of EG concentration for AuNRs alignment. Optimization of the initial AuNRs concentration will be required to precisely control the formation of vertically aligned AuNRs by using inkjet printing. In addition, in the future work, it is important to enable the fabrication of vertically aligned AuNRs with various aspect ratios because the aspect ratio can affect sensing performances.^36^

## Conclusions

In summary, by using an aqueous AuNR dispersion containing a small amount of EG as an ink, we have realized the fabrication of vertically aligned AuNRs based on inkjet printing technology. In this method, the self-assembly process of AuNRs was controlled by utilizing the difference in evaporation rate between EG and water. Therefore, it is expected that it can be applied to the self-assembly of nanoparticles of various shapes such as nanospheres, nanoprisms, and nanocubes. Since an arbitrary pattern can be printed at an arbitrary position on a substrate by using inkjet printing, this work is expected to greatly contribute to the development and practical application of optoelectronic devices and sensors based on self-assembled nanostructures.

## Conflicts of interest

There are no conflicts to declare.

## Supplementary Material

RA-011-D1RA03900H-s001

RA-011-D1RA03900H-s002

RA-011-D1RA03900H-s003

RA-011-D1RA03900H-s004

RA-011-D1RA03900H-s005

RA-011-D1RA03900H-s006

## References

[cit1] Min Y., Akbulut M., Kai K., Golan Y., Israelachvili J. (2008). Nat. Mater..

[cit2] Wang T., Lamontagne D., Lynch J., Zhuang J., Cao Y. C. (2013). Chem. Soc. Rev..

[cit3] Boles M. A., Engel M., Talapin D. V. (2016). Chem. Rev..

[cit4] Fan J. A., Wu C., Bao K., Bao J., Bardhan R., Halas N. J., Manoharan V. N., Nordlander P., Shvets G., Capasso F. (2010). Science.

[cit5] Henzie J., Andrews S. C., Ling X. Y., Li Z., Yang P. (2013). Proc. Natl. Acad. Sci. U. S. A..

[cit6] Liu X., Biswas S., Jarrett J. W., Poutrina E., Urbas A., Knappenberger Jr K. L., Vaia R. A., Nealey P. F. (2015). Adv. Mater..

[cit7] Lin Q.-Y., Li Z., Brown K. A., O'Brien M. N., Ross M. B., Zhou Y., Butun S., Chen P.-C., Schatz G. C., Dravid V. P., Aydin K., Mirkin C. A. (2015). Nano Lett..

[cit8] Wei W., Bai F., Fan H. (2019). Angew. Chem., Int. Ed..

[cit9] Scarabelli L., Hamon C., Liz-Marzan L. (2017). Chem. Mater..

[cit10] Liang Y., Xie Y., Chen D., Guo C., Hou S., Wen T., Yang F., Deng K., Wu X., Smalyukh I., Liu Q. (2017). Nat. Commun..

[cit11] Mubeen S., Lee J., Singh N., Kraemer S., Stucky G., Moskovits M. (2013). Nat. Nanotechnol..

[cit12] Peng B., Li Z., Mutlugun E., Hernandez-Martinez P., Li D., Zhang Q., Gao Y., Demir H., Xiong Q. (2014). Nanoscale.

[cit13] Yin Z., Zhou D., Xu W., Cui S., Chen X., Wang H., Xu S., Song H. (2016). ACS Appl. Mater. Interfaces.

[cit14] Mei Z., Tang L. (2017). Anal. Chem..

[cit15] Martín A., Wang J. J., Iacopino D. (2014). RSC Adv..

[cit16] Peng B., Li G., Li D., Dodson S., Zhang Q., Zhang J., Lee Y., Demir H., Ling X., Xiong Q. (2013). ACS Nano.

[cit17] Milliken S., Fraser J., Poirier S., Hulse J., Tay L.-L. (2018). Spectrochim. Acta, Part A.

[cit18] Wei W., Wang Y., Ji J., Zuo S., Li W., Bai F., Fan H. (2018). Nano Lett..

[cit19] Martin A., Schopf C., Pescaglini A., Wang J. J., Iacopino D. (2014). Langmuir.

[cit20] Liu M., Wang J., He M., Wang L., Li F., Jiang L., Song Y. (2014). ACS Appl. Mater. Interfaces.

[cit21] Gao M., Li L., Song Y. (2017). J. Mater. Chem. C.

[cit22] Rim Y. S., Bae S.-H., Chen H., De Marco N., Yang Y. (2016). Adv. Mater..

[cit23] Kang B., Lee W. H., Cho K. (2013). ACS Appl. Mater. Interfaces.

[cit24] Stüwe D., Mager D., Biro D., Korvink J. G. (2015). Adv. Mater..

[cit25] Kim B. H., Onses M. S., Lim J. B., Nam S., Oh N., Kim H., Yu K. J., Lee J. W., Kim J.-H., Kang S.-K., Lee C. H., Lee J., Shin J. H., Kim N. H., Leal C., Shim M., Rogers J. A. (2015). Nano Lett..

[cit26] Xie Y., Guo S., Guo C., He M., Chen D., Ji Y., Chen Z., Wu X., Liu Q., Xie S. (2013). Langmuir.

[cit27] Jung N., Weon B. M., Doi M. (2020). Soft Matter.

[cit28] Fischer B. J. (2002). Langmuir.

[cit29] Jana N. R., Gearheart L., Murphy C. J. (2001). J. Phys. Chem. B.

[cit30] Liu R., Jiang L., Liu G., Chen G., Li J., Liu J., Wang X.-H. (2019). J. Phys. Chem. C.

[cit31] Liu J., Li J., Li F., Zhou Y., Hu X., Xu T., Xu W. (2018). Anal. Bioanal. Chem..

[cit32] Akbas H., Kartal C. (2006). Colloid J..

[cit33] Yu C., Irudayaraj J. (2007). Anal. Chem..

[cit34] Xu X., Ying Y., Li Y. (2012). Sens. Actuators, B.

[cit35] Frenkel D. (2015). Nat. Mater..

[cit36] Munshi A. M., Ho D., Saunders M., Agarwal V., Raston C. L., Iyer K. S. (2016). Sens. Actuators, B.

