# Supplementary material for: Facile fabrication of self-assembled nanostructures of vertically aligned gold nanorods by using inkjet printing
Source: RSC Adv. 2021 Jun 24;11(36):22376–80. doi: 10.1039/d1ra03900h (PMC9034222; doi:10.1039/d1ra03900h)
Supplement: RA-011-D1RA03900H-s006 [file RA-011-D1RA03900H-s006.pdf]

Electronic Supplementary Material (ESI) for RSC Advances.
This journal is © The Royal Society of Chemistry 2021

Supplementary Information

Facile fabrication of self-assembled nanostructures of vertically aligned gold nanorods by using inkjet printing

Koichiro Saito[1]*, Keegan McGehee[1], Kengo Manabe[1], and Yasuo Norikane[1,2]

[1]Research Institute for Advanced Electronics and Photonics, National Institute of Advanced Industrial Science and Technology (AIST), Tsukuba, Ibaraki 305-8565, Japan

[2]Department of Chemistry, Faculty of Pure and Applied Sciences, University of Tsukuba, Ibaraki, 305-8571, Japan

E-mail: koichiro.saito@aist.go.jp

Materials

For this study, cetyltrimethylammonium bromide (CTAB, 99%), L(+)-ascorbic acid (99.6%), ethylene glycol (EG, 99.5%), silver nitrate ($AgNO_3$, 99.8%), HCl (1.0 M), and $NaBH_4$ (99%), benzyl butyl phthalate (BBP) were all purchased from Fujifilm Wako (Osaka, Japan). $HAuCl_4 \cdot 4H_2O$ (99%) was purchased from Kishida Chemical (Osaka, Japan). All solutions were prepared with ultrapure water (> 18 MΩ ・ cm).

Synthesis of gold nanorods

Synthesis of gold nanorods (AuNRs) was carried out using a CTAB capped seed mediated growth method. Specifically, seed solution is first prepared by addition of 250 μL $HAuCl_4$ (0.01 M) to 9.75 mL CTAB solution (0.1 M). After preparation of a fresh ice cold $NaBH_4$ solution (0.01 M), 600 μL are injected to the seed mixture under rapid stirring. Following several minutes of stirring to remove any potential concentration gradient the seed solution is left to stand for at least 2 hours at 30 ℃.

Growth solutions for the AuNRs synthesis were then prepared. To 40 mL of CTAB solution (0.1M) the following reagents are added sequentially; 2.0 mL $HAuCl_4$ (0.01 M), 400 μL $AgNO_3$

(0.01 M), 800 μL HCl (1.0 M), and 320 μL Ascorbic Acid (0.1 M). Thorough mixing is done after each step to ensure even distribution. 10 μL of matured seed solution are injected into the growth solution. Following gentle mixing, the growth solution is then left to stand at 30 ℃ for at least 16 hours. The synthesized AuNR was centrifuged and redispersed in a 3 mM aqueous CTAB solution. This operation was performed twice to concentrate the concentration of AuNRs to about 3 nM.

Inkjet Printing Process

We used electrostatic inkjet printing (Microjet FemtoJet-2000HB, MICROJET Corp.). The diameter of the nozzle was 40 μm, and the gap between the substrate and the nozzle was 40 μm. AC voltage of 1 kV at 10 Hz was applied between the substrate and the nozzle for 500 ms for each ejection. In order to draw the lines, each droplet was fused by ejecting at intervals of 25 μm. A droplet with a volume of about 100 pL is ejected for each ejection. The width of the line was about 100 μm. The interval between the lines was set to 200 μm. The printed area was 2 mm x 2 mm.

Scanning electron microscopy (SEM) observation

For SEM observation, we used a field emission scanning electron microscope (JSM-6700F, JEOL ltd.). The accelerating voltage was 10 kV and the emission current was 10 μA.

Particle image velocimetry (PIV) analysis

For PIV analysis of water and EG / water mixture, we dropped 0.5 μL of each droplet containing tracer particles (fluorescent pigment, particle size 3~5 μm, JUJO chemical co., ltd.) onto a substrate. The droplet was irradiated with a 532 nm laser (Seika Digital Image corp.), and the motions of the tracer particles were observed with a high-speed camera (FASTCAM Mini AX, Photron co. ltd.). The motions of particles were analyzed using PIV software (KoncertoII, Seika Digital Image corp.). In order to obtain high spatial resolution, multi-grid Interrogation was used as the analysis method.

Surface-enhanced Raman scattering (SERS) measurement

SERS measurements were carried out using a Raman spectrometer (Raman station 400F, Perkin Elmer co., ltd.). Raman spectra were collected in a back-scattering configuration using normal light incidence. Samples were prepared by immersing the substrate with vertically aligned AuNRs in 1 nM BBP ethanol solution for several seconds, then allowing them to dry completely in ambient conditions. Once completely dried the SERS measurement was carried out with 30 collections of 10 s laser exposure (λ = 785 nm). Laser power at the sample was set to 10 mW. The SERS spectrum of a pure BBP liquid was also measured by dropping 10 μL droplets onto Si.

Zeta-potential measurement

The zeta-potentials of the AuNRs were measured by using a zeta-potential measurement system (Zetasizer ZS, Malvern Panalytical co., ltd.) with a 173° scattering angle at a temperature of 25 °C. The cuvettes used in the measurements were disposable DTS 1070 capillary cells. For each AuNRs dispersion, the measurements were carried out three times to calculate the mean and corrected sample standard deviation of the zeta potential.

Supplementary Figure

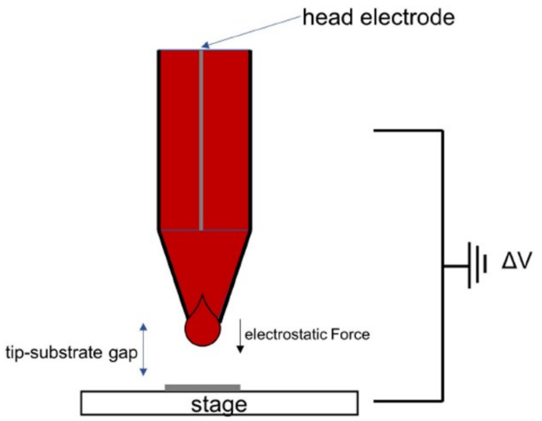

Fig. S1. The electrostatic scheme for inkjet printing. ΔV can be applied with AC current.

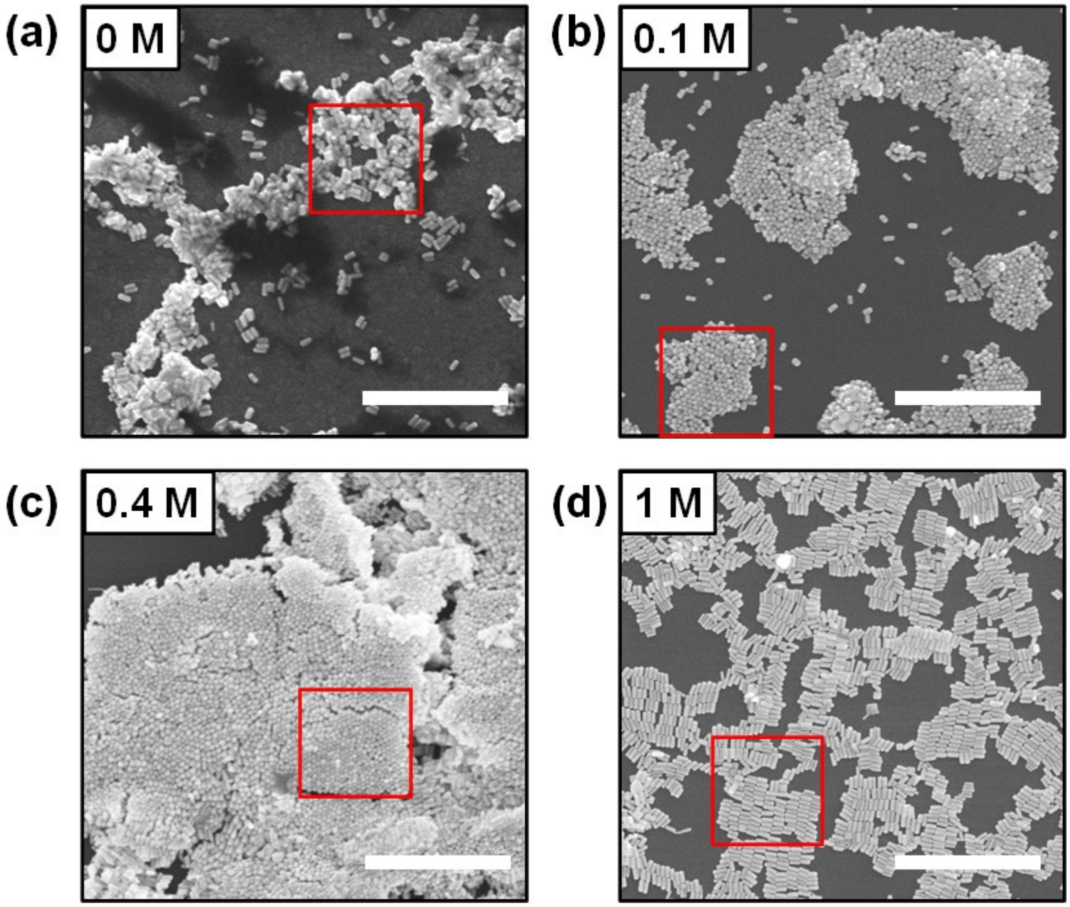

Fig. S2. The nanostructures of AuNRs resulting from varied EG concentration in the printing inks, (a) 0 M, (b) 0.1 M, (c) 0.4 M, and (d) 1.0 M. The scale bars are 1 μm. The squares in the red frame correspond to the images shown in Figure 4 (b).

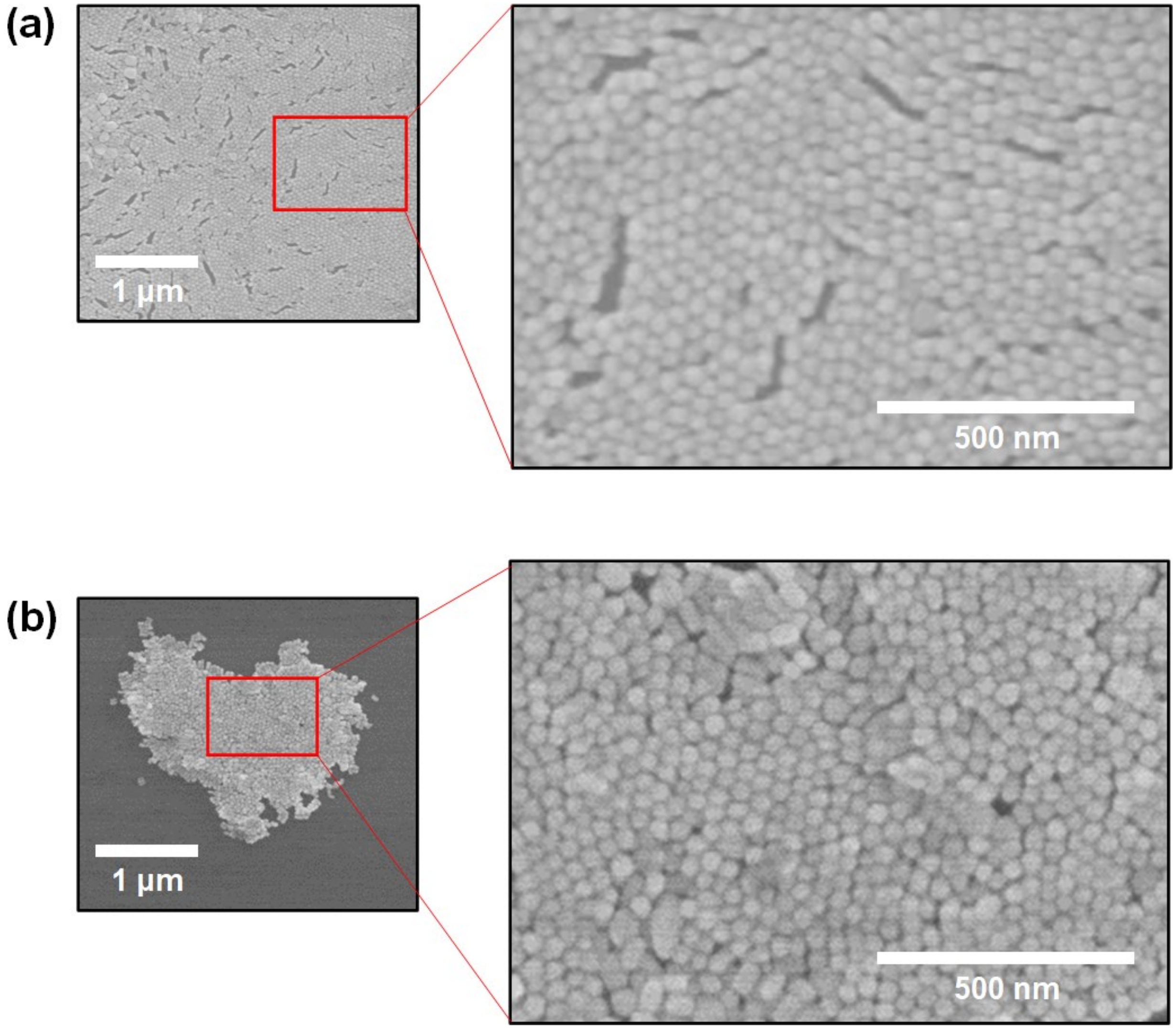

Fig. S3. SEM images of 10 μL dropcasted AuNRs ink droplets for (a) 0.1 M and (b) 0.4 M EG sample.

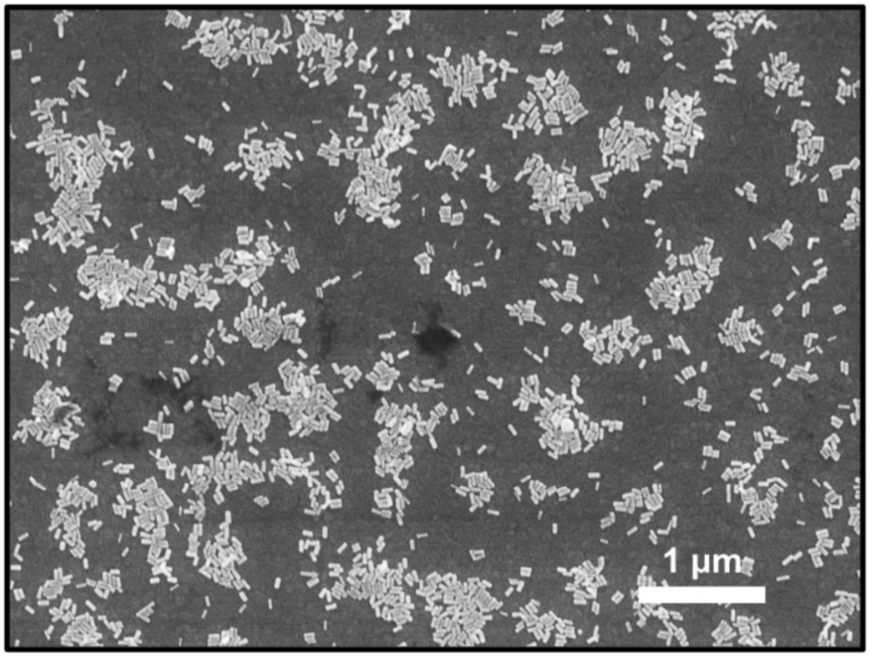

Fig. S4. An SEM image of AuNRs deposited by using inkjet printing for 1.78 M (10 vol%) EG sample.

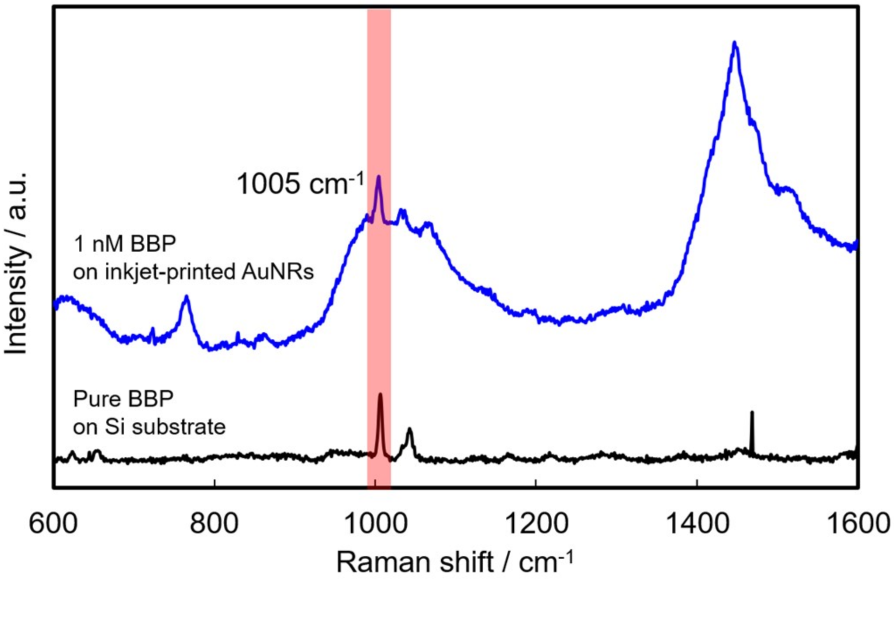

Fig. S5. SERS spectra of 1 nM BBP on vertically aligned AuNRs fabricated by inkjet printing and pure BBP liquid 10 µL on a bare silicon substarte.

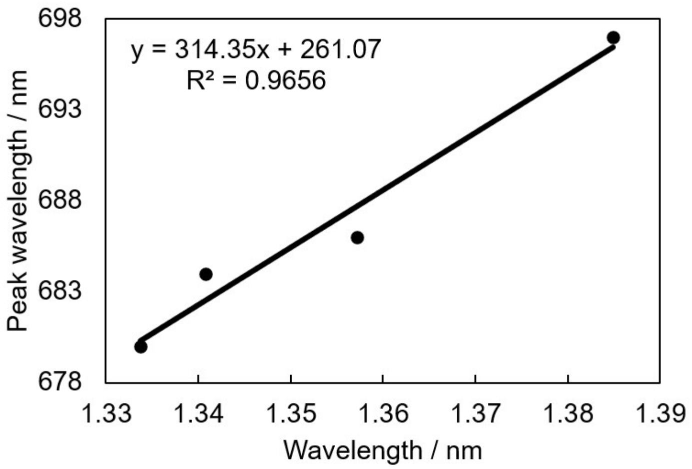

Fig. S6. Linear relationship between the refractive index of the EG/water mixture and the peak wavelength of the AuNRs dispersion. The EG concentrations of 0, 10, 25 and 50 vol% correspond to the refractive indexes of 1.3337, 1.3408, 1.3572 and 1.3849, respectively. The refractive index measurement was carried out by using a digital refractometer (PAL-RI, ATAGO co., ltd.).

Movie Captions

Movie S1. Drying behavior of a droplet of EG/water mixture on a silicon substrate observed by optical microscope. Fast forward speed at 60×real time. (MP4, 2.5 MB)

Movie S2. Drying behavior of a droplet of pure water on a silicon substrate observed by optical microscope. Fast forward speed at 10×real time. (MP4, 1.0 MB)

Movie S3. Drying behavior of a droplet of EG on a silicon substrate observed by optical microscope. Fast forward speed at 300×real time. (MP4, 3.5 MB)

Movie S4. Motion of tracer particles in a droplet of water observed by optical microscope. Arrows which shows the average flow velocity analyzed by PIV are superimposed. (MP4, 6.4 MB)

Movie S5. Motion of tracer particles in a droplet of EG/water mixture observed by optical microscope. Arrows which shows the average flow velocity analyzed by PIV are superimposed. (MP4, 5.8 MB)
